# mAChRs activation induces epithelial-mesenchymal transition on lung epithelial cells

**DOI:** 10.1186/1471-2466-14-53

**Published:** 2014-03-31

**Authors:** Kai Yang, Yun Song, Ya-Bing Tang, Zu-Peng Xu, Wei Zhou, Li-Na Hou, Liang Zhu, Zhi-Hua Yu, Hong-Zhuan Chen, Yong-Yao Cui

**Affiliations:** 1 Department of Pharmacology, Shanghai JiaoTong University School of Medicine, 280 South Chongqing Road, Shanghai 200025, China

**Keywords:** Epithelial-mesenchymal transition (EMT), Lung epithelial cells, Non-neuronal cholinergic system, Signaling pathway

## Abstract

**Background:**

Epithelial-mesenchymal transition (EMT) has been proposed as a mechanism in the progression of airway diseases and cancer. Here, we explored the role of acetylcholine (ACh) and the pathway involved in the process of EMT, as well as the effects of mAChRs antagonist.

**Methods:**

Human lung epithelial cells were stimulated with carbachol, an analogue of ACh, and epithelial and mesenchymal marker proteins were evaluated using western blot and immunofluorescence analyses.

**Results:**

Decreased E-cadherin expression and increased vimentin and α-SMA expression induced by TGF-β1 in alveolar epithelial cell (A549) were significantly abrogated by the non-selective mAChR antagonist atropine and enhanced by the acetylcholinesterase inhibitor physostigmine. An EMT event also occurred in response to physostigmine alone. Furthermore, ChAT express and ACh release by A549 cells were enhanced by TGF-β1. Interestingly, ACh analogue carbachol also induced EMT in A549 cells as well as in bronchial epithelial cells (16HBE) in a time- and concentration-dependent manner, the induction of carbachol was abrogated by selective antagonist of M1 (pirenzepine) and M3 (4-DAMP) mAChRs, but not by M2 (methoctramine) antagonist. Moreover, carbachol induced TGF-β1 production from A549 cells concomitantly with the EMT process. Carbachol-induced EMT occurred through phosphorylation of Smad2/3 and ERK, which was inhibited by pirenzepine and 4-DAMP.

**Conclusions:**

Our findings for the first time indicated that mAChR activation, perhaps via M1 and M3 mAChR, induced lung epithelial cells to undergo EMT and provided insights into novel therapeutic strategies for airway diseases in which lung remodeling occurs.

## Background

Epithelial-mesenchymal transition (EMT) is a process whereby fully differentiated epithelial cells undergo transition to a mesenchymal phenotype, including changes in the expression of epithelial markers, such as E-cadherin, some cytokeratins, and mesenchymal markers, such as vimentin, N-cadherin and α-smooth muscle actin (α-SMA), as well as matrix metallopeptidase 9 (MMP-9) [1,2]. EMT can, therefore, be regarded as a complex manifestation of epithelial plasticity [3].

EMT is increasingly recognized as one of the most important developmental biological processes in normal wound healing. However, dysregulated EMT also appears to occur in the progression and metastasis of cancer as well as the pathogenesis of pulmonary diseases, such as asthma, chronic obstructive pulmonary disease (COPD), and pulmonary fibrosis [4-8]. Transforming growth factor (TGF)-β1 is thought to contribute to EMT and myofibroblast differentiation [9-11]. A recently published report demonstrated, however, that anticholinergic aclidinium inhibits human lung fibroblast to myofibroblast transition induced by TGF-β1 stimulation [12]. Also other reports have found that stimulation of muscarinic acetylcholine receptors (mAChRs) augmented functional TGF-β1 effects in human airway smooth muscle (ASM) cells [13] and TGF-β1-induced Smad activation and ERK phosphorylation in lung fibroblasts was suppressed by anticholinergic tiotropium [14]. These results suggested a potential effect of the non-neuronal cholinergic system in TGF-β1-mediated events. Although AChRs have previously been shown to be potential regulatory role in lung fibroblast to myofibroblast transition, the role of acetylcholine (ACh) which serves as an autocrine or paracrine growth factor in induction of EMT in lung epithelial cells was relatively unexplored.

Airway epithelium presents all components of the cholinergic system, namely muscarinic receptors, ChAT, high-affinity choline uptake, esterase, as well as ACh itself [15,16]. Recently, it was demonstrated that ACh regulates aspects of inflammation and remodeling through its action on AChRs during airway diseases [17-19]. Incubation of lung epithelial cells with ACh resulted in the release of inflammatory mediators. The secretion of these mediators was inhibited by tiotropium, a novel muscarinic antagonist [20]. In epithelial cells derived from COPD patients and smokers, ACh induced a significantly higher release of the inflammatory mediator LTB4 compared to control cells. This release of the lipid mediator was blocked by anticholinergic treatment as well [21]. In a COPD model of LPS-induced airway inflammation and remodeling in guinea pigs, tiotropium abrogated the LPS-induced increase in neutrophils, goblet cells, collagen deposition and muscularised microvessels, but had no effect on emphysema [22]. These results suggested that endogenous acetylcholine plays a major role in the pathogenesis of this disease. EMT takes center stage as the convergence point between inflammation and airway diseases. Inflammatory mediators are known to induce downregulation of epithelial cell–cell adhesion and promote mesenchymal gene expression both in vitro and in vivo, and consequently inflammatory mediators have emerged as decisive factors in EMT induction. Although a number of molecules involved in ACh-mediated airway inflammation and remodeling have been identified, little is known regarding the role of ACh in EMT.

In the current study, we explored (1) whether TGF-β1-induced EMT can be influenced by non-neuronal cholinergic system in lung epithelial cells, and if so, (2) whether mAChR activation has similar effects to TGF-β1 in EMT induction. Moreover, (3) which of the two main pathways, the Smad pathway or the ERK pathway, both of which can be activated by mAChR agonists, was involved in EMT in lung epithelial cells.

## Methods

### Cell culture and treatment

The human alveolar epithelial cell line A549 and human tracheobronchial epithelial cell line 16HBE were obtained from the American Type Culture Collection (Manassas, VA, USA), and were maintained in Ham’s F-12 medium (Gibco, Grand Island, NY, USA) or Dulbecco’s modified Eagle’s medium (DMEM)/HIGH Glucose (HyClone, Logan, UT, USA) supplemented with 10% fetal bovine serum (FBS) and 1% penicillin/streptomycin (Gibco) in a humidified incubator with 5% CO2 at 37°C.

Cells were grown in 96- or 6-well plastic tissue culture plates until confluence and then transferred into serum-free medium containing 0.1% FBS for 24 h. After that, cells were treated as designed.

### Western blot analysis

Cells were lysed in RIPA lysis buffer (50 mM Tris, pH7.4, 150 mM NaCl, 1% Triton X-100, 1% sodium deoxycholate, 0.1% SDS) and a protease inhibitor cocktail (1 mM phenylmethylsulfonyl fluoride, 10 μg/ml pepstatin A, 10 μg/ml aprotinin and 5 μg/ml leupeptin) on ice for 5 min and scraped into a centrifuge tube. The lysates were centrifuged at 13,000 × *g* for 5 min at 4°C. Total protein was mixed with sodium dodecyl sulfate (SDS) sample buffer and heated at 100°C for 5 min. Equal amounts of samples were separated by 10% SDS-polyacrylamide gel electrophoresis and transferred to polyvinylidene fluoride membranes, which were then blocked with 5% non-fat milk in TBS-T (Tris-HCl 50 mM, NaCl 150 mM, Tween-20 0.1%) for 1 h at room temperature (RT) and then incubated with primary antibodies overnight at 4°C. After washing the membranes three times for 5 min each with TBS-T, they were incubated with horseradish peroxidase-conjugated secondary antibodies for 1 h at RT, followed by an additional three washes for 5 min each with TBS-T. Bands were subsequently visualized on film using enhanced chemiluminescence reagents. Results were expressed relative to glyceraldehyde-3-phosphate dehydrogenase (GAPDH) band density used as a loading control. The following antibodies were used: E-cadherin, vimentin, MMP-9 (Cell Signaling Technology, MA, USA), α-SMA (Abcam, MA, USA), GAPDH (Santa Cruz Biotechnology, CA, USA), and ChAT (Millipore Bioscience Research Reagents, CA, USA).

### Determination of ACh by LC–MS/MS

ACh levels in the supernatants of A549 cells were determined by LC–MS/MS as previously described [23].

### Immunofluorescence

Cells were grown on chamber slides and treated as designed. After intermediate washes with cold phosphate-buffered saline (PBS), the cells were fixed with 4.0% paraformaldehyde in PBS for 15 min at RT. The cells were rinsed in cold PBS and blocked in 5% bovine serum albumin for 1 h at RT. The cells were then incubated with primary antibodies overnight at 4°C, washed with cold PBS, incubated with Alexa Fluor-conjugated secondary antibodies at RT for 1 h, washed with PBS again, and then stained with 1 μg/mL (w/v) 4,6-diamidino-2-phenylindole (DAPI) for 5 min at RT. After washing, images were collected using an Axioscope microscope system (Carl Zeiss AG, Jena, Germany) at 40× magnification. The following antibodies were used: E-cadherin (dilution, 1:200), α-SMA (1:200), and vimentin (1:800).

### Measurement of TGF-β1

The amount of TGF-β1 in the supernatants of A549 cells was determined using enzyme-linked immunosorbent assay kits (Rapidbio Lab, West Hills, CA, USA) according to the manufacturer’s instructions.

### Statistics analysis

All data are expressed as mean ± SEM. Data were analyzed by one-way ANOVA (Dunnett’s test) or the Mann-Whitney test where appropriate and statistical significance was accepted at a p-value of < 0.05. Statistical analyses were performed using Prism version 5.0 (Graph-Pad Software, USA).

## Results

### TGF-β1-induced EMT is attenuated by mAChR antagonist

**Figure 1:**
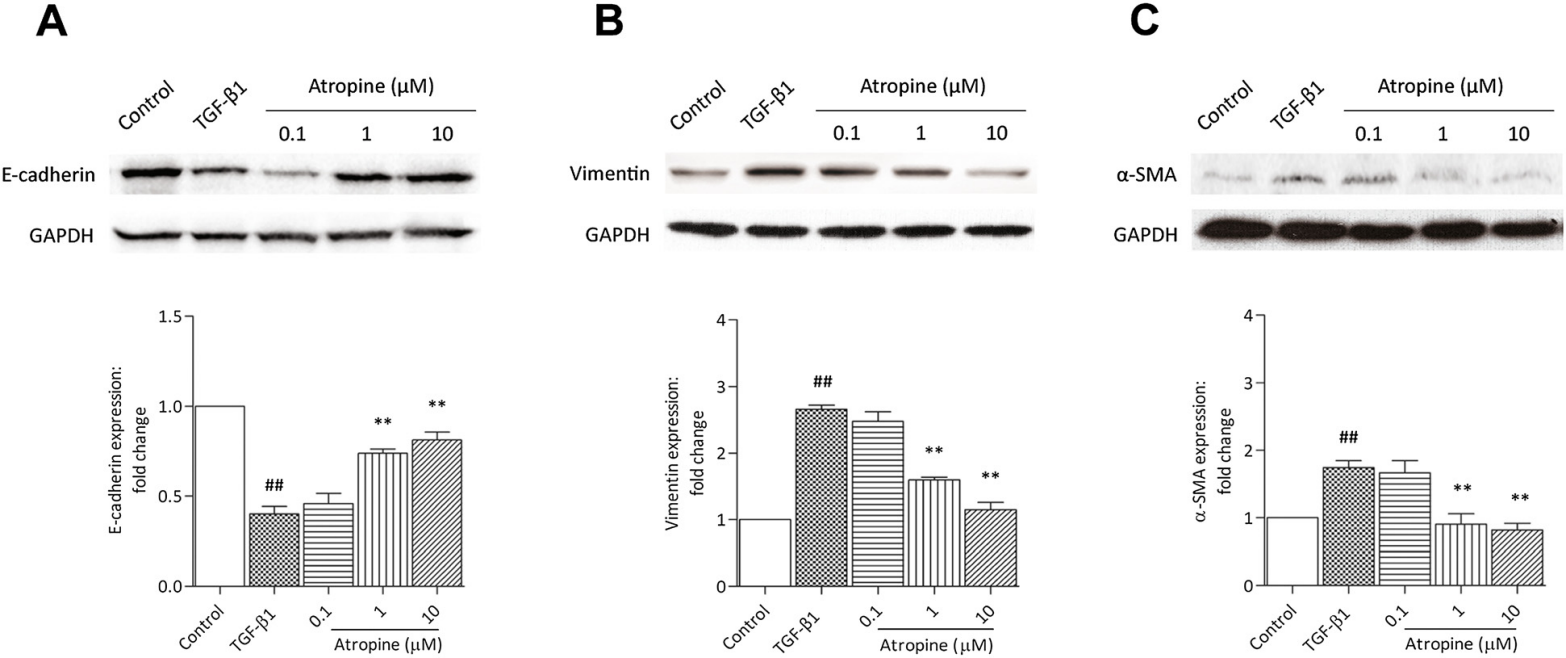
**Effect of mAChR antagonists on TGF-β1-induced EMT in A549 cells.** Cells were treated with atropine (0.1–10 μM) 1 h before stimulation with TGF-β1 (5 ng/mL) for 72 h. Cell lysates were assayed for EMT markers and GAPDH. Representative western blots of E-cadherin **(A)**, vimentin **(B)**, α-SMA **(C)** and GAPDH are shown. Data are expressed as mean ± SEM of 3–5 independent experiments after densitometric analysis. ^##^*p* < 0.01 vs. control; **p < 0.01 vs. TGF-β1.

EMT is defined by changes in gene expression in which epithelial markers are decreased while mesenchymal markers are increased. We examined whether TGF-β1-induced EMT events could be modulated by mAChRs in lung epithelial cells. As expected, A549 cells exposed to TGF-β1 for 72 h resulted in a decrease in the epithelial marker E-cadherin (*p* < 0.01), as well as an increase in the mesenchymal markers vimentin (*p* < 0.01) and α-SMA (*p* < 0.01) (Figure 1A, B, C). Interestingly, TGF-β1-induced EMT events were significantly arrested by the non-selective mAChR antagonist atropine in a dose-dependent manner (0.1–10 μM) (Figure 1A, B, C). This result suggested a modulatory effect of mAChRs and prompted us to surmise a potential effect of endogenous ACh in EMT induction.

### TGF-β1- induced EMT is modulated by non-neuronal cholinergic system

**Figure 2:**
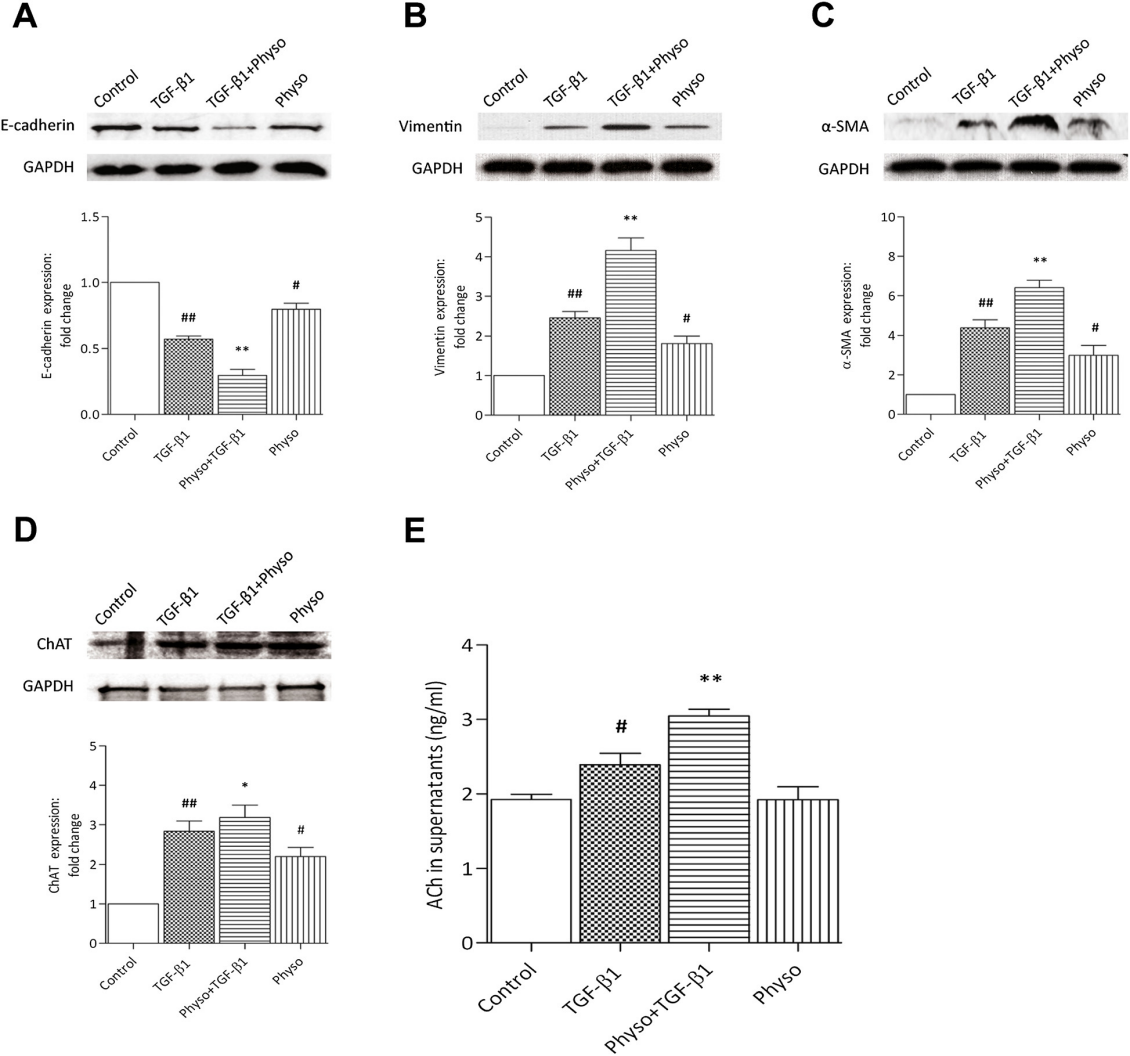
**TGF-β1-induced EMT is modulated by non-neuronal cholinergic system.** Physostigmine (Physo, 100 μM) was added 1 h before stimulation with TGF-β1 (5 ng/mL) for 72 h. ACh in supernatants was determined with an LC–MS/MS assay. Cell lysates were assayed for EMT markers, ChAT and GAPDH. Representative western blots of E-cadherin **(A)**, vimentin **(B)**, α-SMA **(C)**, ChAT **(D)** and GAPDH are shown. **E**: ACh level. Data are expressed as mean ± SEM of 4-6 independent experiments. ^#^*p* < 0.05, ^##^*p* < 0.01 vs. control; *p < 0.05, **p < 0.01 vs. TGF-β1.

To further assess the potential effect of endogenous ACh in EMT events in A549, the acetylcholinesterase (AChE) inhibitor physostigmine was used to increase the amount of ACh by blocking ACh degradation. Interestingly, however, a significant, additive effect was observed by the combined administration of physostigmine and TGF-β1 at 72 h post-stimulation. The change in the expression of TGF-β1-induced E-cadherin, vimentin, and α-SMA was significantly enhanced (*p* < 0.01) by physostigmine (100 μM) compared with TGF-β1 alone (Figure 2A, B, C). In addition, the EMT event also occurred in the presence of physostigmine alone compared with controls (*p* < 0.01) (Figure 2A, B, C).

Having observed the effect of AChE inhibitor on EMT process, we went on to measure ACh levels in the supernatants of cultured A549 cells. This was evaluated by western blot analysis. The ChAT is key enzyme for ACh synthesis and, therefore, the expression of ChAT supernatant levels definitively demonstrated to prove the existence of the endogenous ACh. As shown in Figure 2D, high level expression of ChAT was observed in A549 cells stimulated by TGF-β1, and TGF-β1-induced ChAT expression was enhanced by physositigmine. To further determine if A549 cells express the ChAT needed for ACh synthesis and release, LC-MS/MS were performed. As shown in Figure 2E, in non-stimulated cells, the ACh levels in the culture supernatants were close to the assay’s limit of detection (1.93 ± 0.09 ng/ml). The addition of physostigmine to non-stimulated A549 cell cultures was not associated with a significant increase in ACh levels (1.90 ± 0.15 ng/ml), which were close to the limit of detection. However, the ACh could be readily detected in the presence of TGF-β1 with a significant increase in ACh levels (2.39 ± 0.16 ng/ml). Physostigmine enhanced TGF-β1-induced ACh release by 28%, when compared with TGF-β1 alone (TGF-β1 + physostigmine: 3.07 ± 0.07 ng/ml). Thus, these findings demonstrate that ChAT express and ACh release by A549 cells were enhanced by TGF-β1, and the levels of ACh are modulated by AChE.

### Carbachol induces EMT-related changes in lung epithelial cells

**Figure 3:**
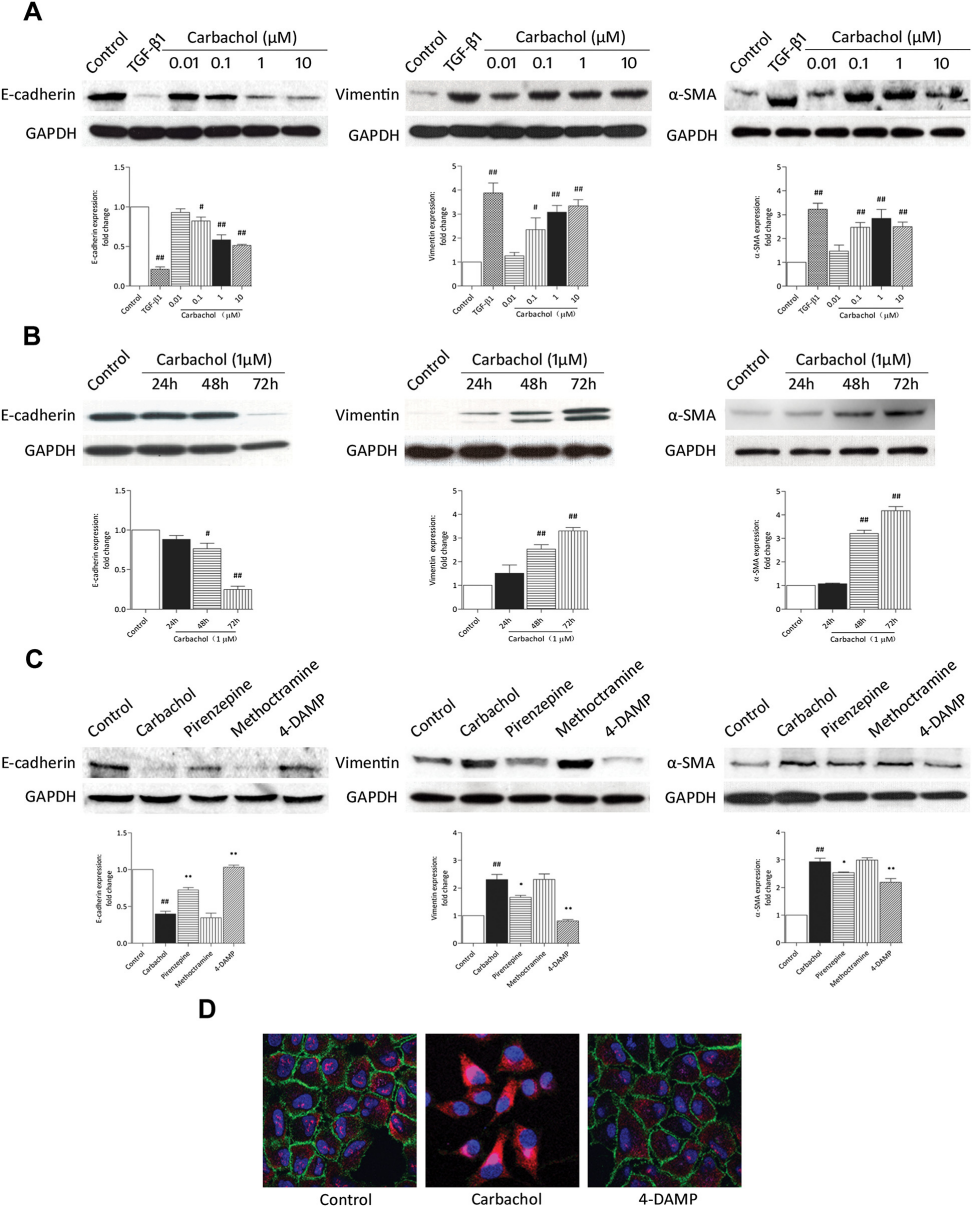
**Carbachol induces EMT-related changes in A549 cells.** Cells were stimulated with TGF-β1 (5 ng/mL) or carbachol (0.1–10 μM) for 72 h **(A)**, carbachol (1 μM) for the indicated times **(B)**, or with the addition of pirenzepine (10 μM), methoctramine (10 μM), or 4-DAMP (1 μM) administered 1 h before carbachol (1 μM) for 72 h **(C)**. Cell lysates were assayed for EMT markers and GAPDH. Representative western blots of E-cadherin, vimentin, α-SMA and GAPDH are shown. **(D)** Cells were treated with 4-DAMP (1 μM) 1 h before stimulation with carbachol (1 μM) for 72 h. Cells were fixed and stained with E-cadherin, vimentin and α-SMA antibodies, and then incubated with Alexa Fluor-conjugated secondary antibodies and DAPI for nuclei labeling. E-cadherin (green), vimentin (purple), α-SMA (red) and nuclei (blue) were visualized using confocal fluorescence microscopy. Data are expressed as mean ± SEM of 3–5 independent experiments after densitometric analysis. ^#^*p* < 0.05, ^##^*p* < 0.01 vs. control; *p < 0.05, **p < 0.01 vs. carbachol.

If endogenous ACh is involved in TGF-β1-induced EMT, the application of an exogenous mAChR agonist should have the same effect as endogenous ACh. As shown in Figure 3A, B, C, carbachol dramatically decreased E-cadherin expression, and increased expression of vimentin and α-SMA in A549 cells in a concentration-dependent manner (Figure 3A). The expression levels of E-cadherin, vimentin and α-SMA significantly changed at 48 h and peaked at 72 h (Figure 3B). It is interesting to note that carbachol at concentrations as low as 0.1 μM was sufficient to induce EMT phenotypic markers with a maximal response at 10 μM. Furthermore, carbachol-induced EMT can be abrogated by pirenzepine (M1 mAChR antagonist, 10 μM) and diphenyl-acetoxy-4-methylpiperidine methiodide (4-DAMP, an M3 mAChR antagonist, 1 μM), but not methoctramine (an M2 mAChR antagonist, 10 μM) (Figure 3C).

To further confirm changes in E-cadherin, vimentin, and α-SMA, immunofluorescence analysis was performed to assess the roles of carbachol on these markers in A549 cells. Confocal laser scanning microscopy images in untreated control cells revealed localized expression of the epithelial marker E-cadherin at cell borders and relatively low expression of the mesenchymal markers vimentin and α-SMA (Figure 3D). Stimulation with 1 μM carbachol for 72 h reduced membrane-associated expression of E-cadherin with loss of expression at cell borders and concomitant dramatic increases in expression of vimentin and α-SMA in contrast to untreated control cells, and these effects were reversed by the mAChR antagonist 4-DAMP (Figure 3D).

**Figure 4:**
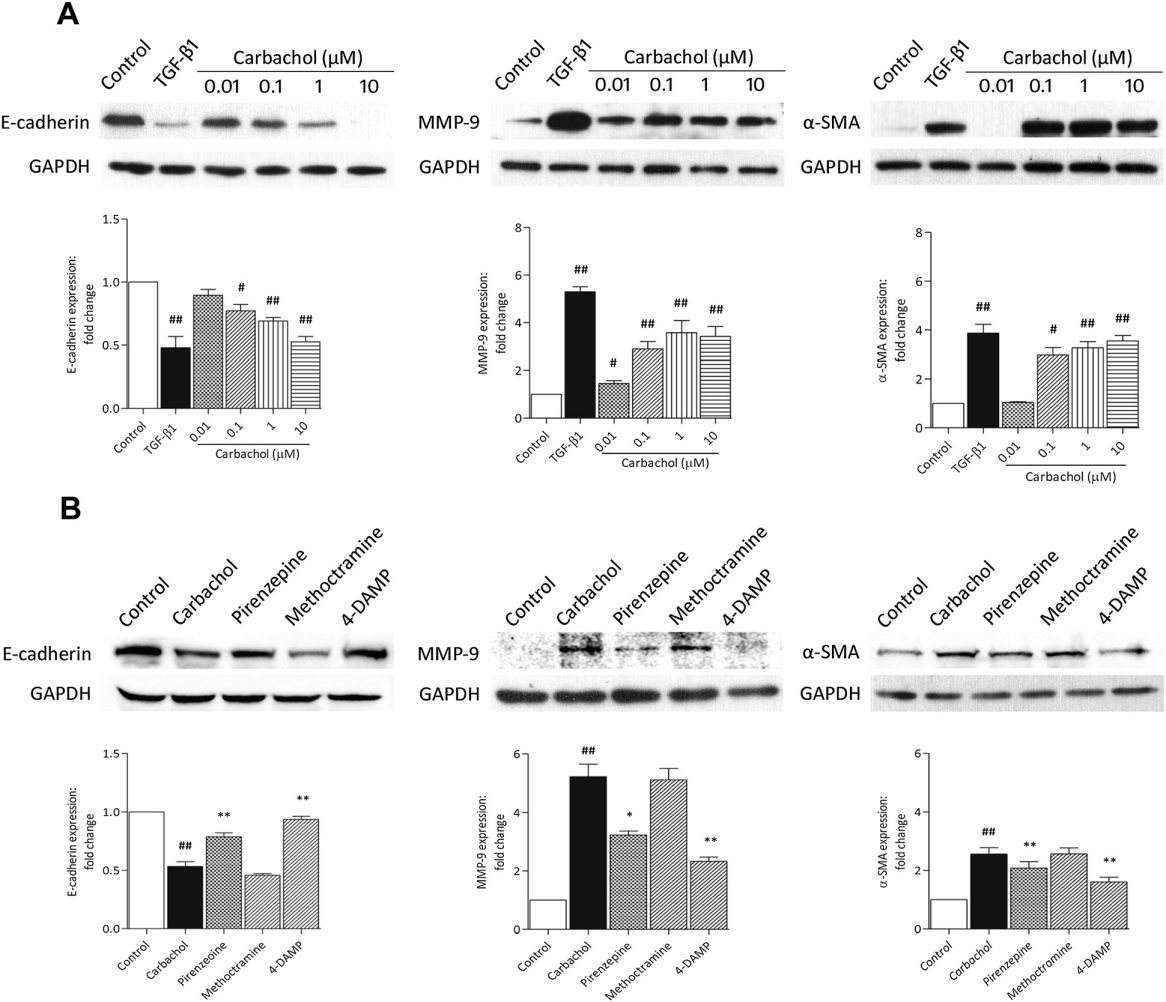
**Carbachol induces EMT-related changes in 16HBE cells.** Cells were stimulated with TGF-β1 (5 ng/mL) or carbachol (0.1–10 μM) for 72 h **(A)** or with the addition of pirenzepine (10 μM), methoctramine (10 μM), or 4-DAMP (1 μM), which was administered 1 h before carbachol (1 μM) for 72 h **(B)**. Cell lysates were assayed for EMT markers and GAPDH. Representative western blots of E-cadherin, MMP-9, α-SMA, and GAPDH are shown. Data are expressed as mean ± SEM of six independent experiments after densitometric analysis. ^#^*p* < 0.05, ^##^*p* < 0.01 vs. control; *p < 0.05, **p < 0.01 vs. carbachol.

To ensure that these findings were not unique to A549 cells, we performed parallel experiments using the human bronchial epithelial cell line 16HBE to assess whether bronchial epithelial cells also undergo EMT during carbachol stimulation. Western blot analysis revealed that E-cadherin expression was decreased in the same manner as in A549 cells, whereas MMP-9 and α-SMA expression in 16HBE cells was increased by carbachol treatment (Figure 4A). The effect of carbachol was significantly inhibited by pirenzepine (10 μM) and 4-DAMP (1 μM), but not methoctramine (10 μM) (Figure 4B).

### Carbachol-induced EMT related to TGF-β1 release from A549 cells

**Figure 5:**
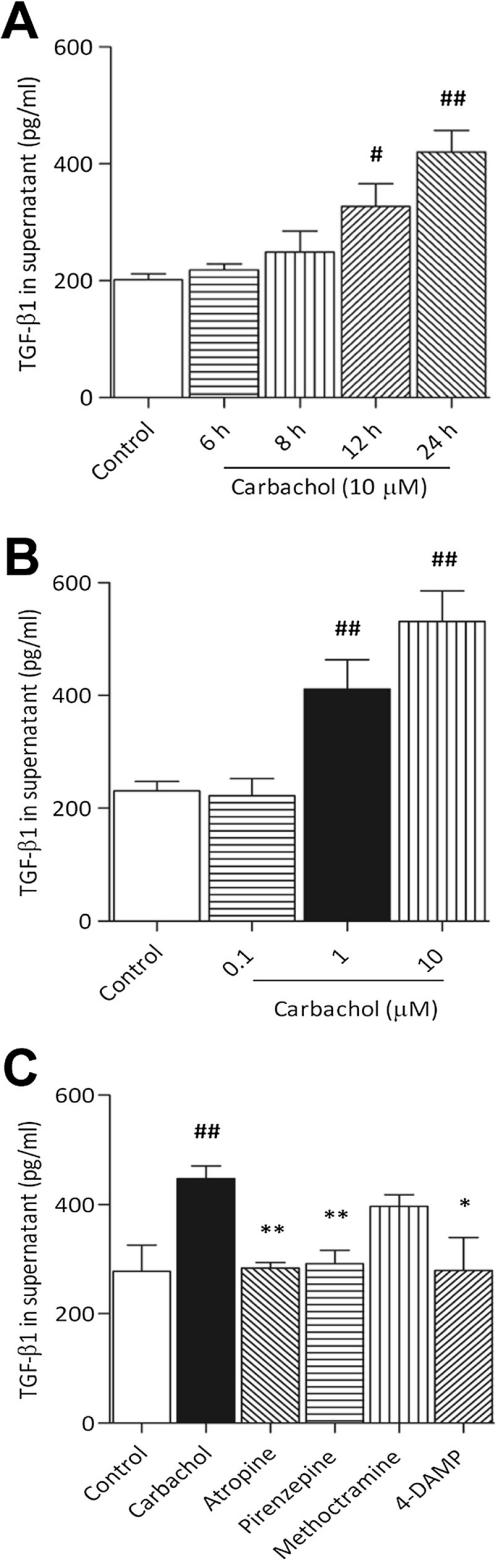
**Effect of carbachol on TGF-β1 release by A549 cells.** Cells were stimulated with carbachol (10 μM) for the indicated times **(A)** or carbachol (0.1–10 μM) for 24 h **(B)**, or with the addition of atropine (10 μM), pirenzepine (10 μM), methoctramine (10 μM), or 4-DAMP (1 μM), which was administered 1 h before stimulation with carbachol (1 μM) for 24 h **(C)**. Data are expressed as mean ± SEM. from at least four experiments. ^#^*p* < 0.05, ^##^*p* < 0.01 vs. control; *p < 0.05, **p < 0.01 vs. carbachol.

We next investigated whether carbachol-induced EMT was related to TGF-β1 expression. To this aim, we stimulated A549 cells for 24 h with carbachol and analyzed EMT events. We found that carbachol induced TGF-β1 production in the supernatant of A549 cells in a time- (6–24 h) and concentration- (0.1–10 μM) dependent manner (Figure 5A and B). Moreover, carbachol-induced TGF-β1 expression was completely abrogated by atropine (10 μM), pirenzepine (10 μM), and 4-DAMP (1 μM) (Figure 5C). These findings suggested that carbachol-induced EMT may be, in part, due to TGF-β1, and cooperative regulation in EMT by mAChR activation and TGF-β1 expression.

### Involvement of the Smad and ERK pathways in carbachol-induced EMT

**Figure 6:**
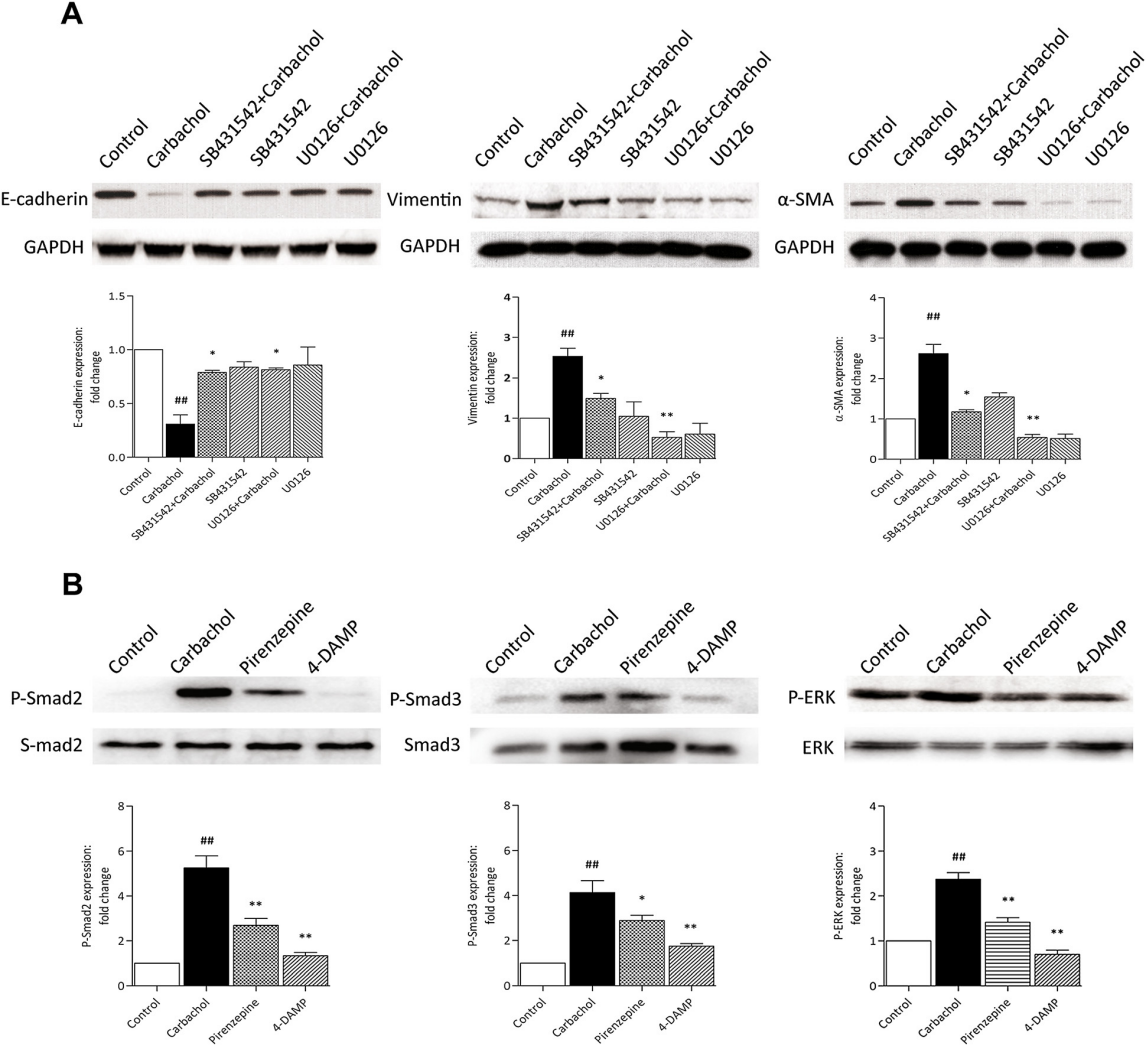
**The involvement of the Smad and ERK pathways in carbachol-induced EMT in A549 cells.** The TGF-β/Smad inhibitor (SB431542, 10 μM), the ERK inhibitor (U0126, 5 μM), pirenzepine (10 μM), or 4-DAMP (1 μM) was administered 1 h before stimulation with carbachol (1 μM). Cell lysates were assayed for EMT markers and GAPDH after incubation for 72 h **(A)**. The expression of Smad2/3/p-Smad2/3 and ERK/p-ERK were detected after incubation for 1 h and 72 h, respectively. **(B)**. Representative western blots are shown. Data are expressed as mean ± SEM of 4–6 independent experiments after densitometric analysis. ^#^*p* < 0.05, ^##^*p* < 0.01 vs. control; *p < 0.05, **p < 0.01 vs. carbachol.

To confirm whether the Smad and ERK pathways, both of which can be activated by mAChR agonists, were involved in carbachol-induced EMT in A549 cells, pharmacological inhibitors were used to inhibit each pathway. We found that carbachol-induced EMT was completely inhibited by addition of the TGF-β/Smad inhibitor SB431542 (10 μM) and the ERK inhibitor U0126 (5 μM) (Figure 6A). Moreover, both Smad2/3 and ERK phosphorylation induced by 1 μM carbachol were significantly inhibited by 10 μM pirenzepine and 1 μM 4-DAMP (Figure 6B). These findings indicated that both the Smad2/3 and ERK signaling pathways were involved in carbachol-induced EMT and mAChR activation, perhaps M1 and M3 mAChRs induce downstream target gene expression during the EMT process.

## Discussion

Our work revealed that TGF-β1-induced EMT in lung epithepial cells can be abrogated by mAChR antagonist and enhanced by the AChE inhibitor, and that ACh synthesis and release from lung epithelial cells can be enhanced by TGF-β1. In addition, mAChR stimulation with ACh analogue carbachol also induced lung epithelial cells to undergo EMT. Our findings demonstrated that non-neuronal cholinergic system components involved in EMT in lung epithelial cells and provided insights into novel therapeutic strategies for airway diseases in which lung remodeling occurs.

Several studies have reported increased TGF-β expression in the airway epithelium of patients with obstructive airway diseases [9,24]. Furthermore, there is much evidence that TGF-β1 is a primary regulator of EMT [25,26]. The pulmonary alveolar surface is lined with type I and type II epithelial cells. Type II cells are since long recognized as important players of the innate immune system, producing cytokines and chemokines. The cancer-derived human alveolar epithelial cell line A549 is widely acknowledged as a relevant model of type II alveolar epithelial cells and the ability to undergo EMT in vitro has been confirmed [10]. We also observed an almost identical EMT pattern following stimulation with carbachol in 16HBE cells. As a result, carbachol-induced EMT events were not limited to alveolar epithelial cells, but also extended to bronchial epithelial cells in vitro, although there were differences in the expression of the typical mesenchymal markers vimentin and MMP-9 between A549 and 16HBE cells. This difference in expression profiles might have been due to variances between the cells types investigated. The present findings were in accordance with other studies in which TGF-β1 reduced E-cadherin mRNA levels while simultaneously increasing expression of α-SMA and MMP-9, but not vimentin, in human bronchial epithelial cells (BEAS-2B) [27], and TGF-β1 had almost no effect on MMP-9 expression in the A549 cell line [10].

Epithelial cells can express the machinery of the non-neuronal cholinergic system, comprising ACh-synthesizing choline acetyltransferase, the vesicular ACh transporter, nicotinic ACh receptors, mAChRs, and the ACh-hydrolyzing enzymes acetylcholinesterase and butyrylcholinesterase [28-30]. The cells were able to synthesize and release ACh [20,23,31] and could also be activated by ACh itself. Of the five molecular subtypes of mAChR, three (M1 mAChR, M2 mAChR, and M3 mAChR) reportedly mediate distinct physiological functions in the lung.

In our present study, we found that TGF-β1-induced EMT could be modulated by mAChR antagonists and that A549 cells stimulated with TGF-β1 synthesize and secrete ACh, suggesting a potential effect of endogenous ACh in EMT induction. Further studies supported the idea that the ACh analog carbachol induced EMT resulting in dramatic down-regulation of E-cadherin, and up-regulation of vimentin and α-SMA in lung epithelial cells. Similar findings were described in the transition of human lung fibroblasts to myofibroblasts [12]. Interestingly, low doses of carbachol (0.1 μM) induced loss of epithelial marker expression in A549 cells and concurrent gains in mesenchymal markers. The data obtained in the present study extend and reinforce our previous speculations and showed that the cellular switch from an epithelial to mesenchymal-like phenotype may be occurred in lung epithelial cells and triggered by endogenous ACh secreted by A549 cells. Also, in accordance with our previous findings, the effect of physostigmine alone and in combination with TGF-β1, this was able to upregulate choline acetyltransferase expression in A549 cells. Therefore, we reasoned that the physostigmine-related EMT event observed in the present study increased the amount of ACh by blocking ACh degradation and activating mAChRs in A549 cells. Importantly, epithelial cells (e.g., A549 cells) express all of the necessary components to synthesize and release ACh by themselves, which acts as an autocrine growth factor via mAChR activation [32].

Recent studies have revealed that in addition to inflammation, ACh regulates important aspects of lung structure via the M1 or M3 mAChR pathways. Indeed, M1 or M3 mAChRs are both expressed by structural cells of the airways, including epithelial cells and ASM cells [23,33]. Moreover, in vitro studies have demonstrated a role for M1 or M3 mAChR in the regulation of ASM proliferation [34]. In our study, we found that carbachol-induced EMT can be abrogated by M1 or M3 mAChR-selective antagonists. In fact, the involvement of mAChRs in carbachol-induced EMT supported the finding that the EMT process might be modified by M1 and M3 mAChR antagonists acting on lung epithelial cells. This finding was in accordance with the results reported by Milara et al., which showed that M1 and M3 mAChRs were involved in carbachol- or TGF-β1-induced fibroblast to myofibroblast transition in human lung fibroblasts [12].

Since both carbachol and TGF-β1 can induce EMT via epithelial to mesenchymal transition, an interaction between mAChRs and TGF-β1 in EMT induction may also be expected. Kong et al. found a cooperative regulation by G protein-coupled receptor ligands and growth factors [35]. Recently, a strong relationship between mAChRs and TGF-β1 has been illustrated, and carbachol stimulation has been reported to increase TGF-β1 expression [12]. However, emerging evidence suggests that an interaction of mAChR activation and TGF-β1 expression may contribute to EMT induction. The results of the present study suggested that (1) TGF-β1-induced EMT can be inhibited by mAChR antagonists, (2) mAChR activation induced TGF-β1 expression in A549 cells, and (3) TGF-β1-induced EMT was enhanced by AChE inhibitor which increased the amount of ACh, and lung epithelial cells synthesize and secrete ACh in response to TGF-β1. Thus, the interaction between mAChRs and TGF-β1 in EMT induction can be described as follows: mAChR activation amplifies the signaling pathways governing TGF-β1-mediated EMT events as a result of enhanced EMT processes. This finding was unexpected and suggested that cooperative regulation by mAChR activation and TGF-β1 was involved in EMT, leading to airway remodeling.

Accumulating evidence has indicated that, in addition to Smad2-mediated pathways [3,10,36], other pathways, such as the p38, ERK, c-Jun N-terminal kinase, and mitogen-activated protein kinase pathways have been implicated in TGF-β signaling [14,37]. In the present study, we provide new evidence on the mechanism by which carbachol increases the release of the TGF-β1, the phosphorylation of Smad2/3 and ERK, thus promoting the EMT process in lung epithelial cells. These findings extend and reinforce other report from human bronchial fibroblasts that TGF-β1 activated non-neuronal cholinergic system [12]. Furthermore, we observed that mAChRs antagonist (M1 and M3) suppressed the release of TGF-β1 and the phosphorylation of Smad2/3 and ERK which activated by carbachol resulting in suppression of EMT process. Collectively, these findings suggested that the Smad2/3 and ERK signaling pathways involved in EMT were trigged by mAChR agonists and that a crosstalk of the ERK and TGF-β signaling pathways may potentiate and synergize the canonical TGF-β-Smad pathway, although further work is obviously needed to rule out the effects of other signaling pathways.

## Conclusion

In summary, our results point towards a crosstalk between mAChR activation and TGF-β1 expression in EMT induction in lung epithelial cells and demonstrated that lung epithelial cells secreting ACh may function as an autocrine growth factor via activation of M1 and M3 mAChRs to induce EMT through the Smad2/3 and ERK signaling pathways. These findings demonstrated for the first time the role of non-neuronal cholinergic system in EMT and provided insights into novel therapeutic strategies for airway diseases in which lung remodeling occurs.

## Competing interests

The authors have no competing interests (financial or otherwise) with respect to this article.

## Authors’ contributions

YK and SY carried out the biochemical studies, participating in the western blot experiments and drafting the manuscript. ZW and HLN carried out the immunoassays, participating in ELISA test and immunofluorescence analysis. TYB participated in the LC-MS experiment. YZH participated in the cell cultures. ZL and XZP participated in the design of the study and performed the statistical analysis. CHZ and CYY conceived of the study, and participated in its design and coordination and helped to draft the manuscript. All authors read and approved the final manuscript.

## Pre-publication history

The pre-publication history for this paper can be accessed here:

http://www.biomedcentral.com/1471-2466/14/53/prepub
